# The relationship between personal COVID-19 vaccination decisions and cattle vaccination practices: Evidence from a survey of South Dakota beef producers

**DOI:** 10.1371/journal.pone.0349234

**Published:** 2026-05-18

**Authors:** Filip Viskupič, Russell Daly, David L. Wiltse, Michael Gonda

**Affiliations:** 1 Department of Political Science, Iowa State University, Ames, Iowa, United States of America; 2 Department of Veterinary and Biomedical Sciences, South Dakota State University, South Dakota, United States of America; 3 School of American and Global Studies, South Dakota State University, South Dakota, United States of America; 4 Department of Animal Science, South Dakota State University, South Dakota, United States of America; Central Laboratory for Evaluation of Veterinary Biologics, Agricultural Research Center, EGYPT

## Abstract

**Objectives:**

Vaccination was a contentious issue during the COVID-19 pandemic, and experts warned about a possibility of a spillover effect—negative attitudes toward COVID-19 vaccines might start affecting attitudes toward other vaccines. We evaluated the correlates between beef producers’ personal vaccination decisions and cattle vaccination practices.

**Methods:**

Using publicly available information from USDA payment program participants, we invited 6,238 beef producers in South Dakota to participate in an online survey. The survey was conducted in October 2024 and measured the use of established cattle vaccines, attitudes toward a cattle vaccine for Highly Pathogenic Avian Influenza (HPAI), and personal COVID-19 vaccine uptake. Multivariate logistic regression was utilized to analyze the data.

**Results:**

We received responses from 339 beef producers. We found that 94% of participants regularly vaccinate their cattle with established vaccines, while 24% were likely to vaccinate their cattle for HPAI when a vaccine becomes available. Over 44% of respondents never received a COVID-19 vaccination. Regression analysis results showed no association between COVID-19 vaccine uptake and the use of established cattle vaccines (aOR: 1.45, 95% CI: 0.76–2.74) and a positive and statistically significant association with the likelihood of using HPAI vaccine in cattle (aOR: 1.78, 95% CI: 1.41–2.26).

**Conclusion:**

Overall, we found partial evidence for the presence of a relationship. It is plausible that beef producers who have not received a COVID-19 vaccination might be less likely to adopt new cattle vaccines in the future.

## Introduction

The sustainability of beef cow-calf operations depends upon the efficient production of healthy, productive calves. In addition to proper management of cow and calf nutrition, reproductive parameters, and the environments inhabited by these animals, disease prevention is critical to optimal herd performance. Infectious diseases have the potential to diminish reproductive success and contribute to illness and mortality in breeding-age animals and calves. Vaccination programs are utilized by cow-calf producers to mitigate the effects of these infectious diseases [1].

Cattle producers have been using vaccines – in some form – for disease prevention and control for at least the past 100 years [2]. Common diseases and conditions for which vaccines are used include infectious respiratory disease pathogens that cause Bovine Respiratory Disease in calves after weaning and infectious reproductive pathogens that adversely affect reproductive processes in cows and bulls. Pathogens that animals may encounter in their environments can cause conditions such as anthrax, blackleg, and neonatal diarrhea [1].

In recent years, the outbreak of highly pathogenic avian influenza (HPAI) has presented a threat to the health of beef cattle. In 2015, HPAI was detected in wild bird species in South Dakota, affecting domestic poultry operations (9 flocks with 1.7 million birds total). The current HPAI outbreak beginning in 2022 has affected over 1,700 commercial as well as backyard flocks with more than 174 million birds affected [3]. In spring 2024, the HPAI virus began affecting dairy cattle in numerous states, including 7 herds in South Dakota. While HPAI infections in domestic poultry featured high mortality and euthanasia of remaining birds on affected farms, infected dairy cows exhibited transient (although profound) drops in milk production and increases in secondary infections and other conditions. Moreover, dairy herds affected by HPAI have shown relatively low incidence rates of infection, reported by most to be in the 10–15% range [4]. Although HPAI infections have not yet been documented in beef cattle, beef breeds can be infected with other influenza viruses, so it is not unreasonable to expect that beef cattle may also be susceptible to HPAI [5,6].

Scientists have started developing HPAI vaccines for cattle. In October 2024, vaccine companies began conducting HPAI vaccine field trials. The vaccines will have to go through the approval process before they become available for commercial use. Besides development and authorization, the acceptance by cow-calf producers presents another challenge to the use of these cattle vaccines. In addition to access and costs, producers need to have positive attitudes toward these vaccines. If concerns exist about the safety or effectiveness of these cattle vaccines, it is likely that some producers will be reluctant to use these HPAI vaccine for their cattle. Negative attitudes held by producers might lead to lower uptake and leave livestock vulnerable to the virus.

The development and field testing of the HPAI vaccine for cattle has taken place in the post-COVID-19 pandemic period, characterized by heightened vaccine skepticism and lowered vaccine confidence. COVID-19 vaccination became a salient and, at the same time, sensitive and emotionally charged topic following the outbreak of COVID-19. It is well documented that some people, particularly residents of rural areas and people who work in the agricultural sector, developed negative attitudes toward COVID-19 vaccines and did not get vaccinated [7,8]. Scholars expressed concern about the possibility that people’s negative attitudes toward COVID-19 vaccines might affect their attitudes toward other vaccines [9]. They warned about a potential “spillover effect” happening in the post COVID-19 pandemic period, and those with negative attitudes toward COVID-19 vaccines will show negative attitudes toward other vaccines [10,11].

Research indicates that negative attitudes toward COVID-19 vaccines are affecting attitudes toward other vaccines, such as flu vaccines or hypothetical vaccines [11,12]. Some evidence even suggests an effect on animal vaccines. Studies showed that pet owners with negative attitudes toward human vaccines, including COVID-19 vaccines, were less likely to vaccinate their pets [13,14].

It is possible that attitudes toward COVID-19 vaccines might affect beef producers’ use of cattle vaccines. Those producers who did not receive a COVID-19 vaccination might become less likely to use HPAI vaccines for their cattle. Unlike pet vaccination, vaccinating cattle is a business decision as beef producers depend financially on raising and selling healthy cattle. One might argue that their own health behavior, including COVID-19 vaccination, should be unrelated to cattle vaccination decisions. We explored the relationship between personal vaccine uptake and animal vaccination practices among beef producers who raise beef cattle for commercial purposes.

We conducted a survey of South Dakota beef producers at the time companies started field trials for HPAI vaccine for cattle. This study had two goals. First, we investigated beef producers’ use of established cattle vaccines and attitudes toward HPAI vaccines for cattle. Second, we explored if there was a relationship between beef producers’ personal vaccination decisions and cattle vaccination practices, which has been understudied. We examined the use of established cattle vaccines and the likelihood of using a HPAI vaccine for cattle. The findings will contribute to the scholarly literature in the veterinary medicine and public health fields and inform professionals in veterinary science and animal vaccine manufacturers.

## Methods

### Participants and procedures

The data came from a cross-sectional survey of beef producers from South Dakota, which was developed and fielded by the authors. Using the publicly available 2022 report from the United States Department of Agriculture (USDA) on all Farm Service Agency (FSA) program recipients in the state, we identified potential beef producers in South Dakota. To focus our sample on livestock producers, we consulted with the local FSA office to identify programs that most commercial beef producers would participate in. In sum, nine separate programs commonly associated with cattle and livestock production were identified. These programs were: Livestock forage program, livestock indemnity program, emergency assistance for livestock, pandemic livestock indemnity program, farm ranchers program, trade mitigation program for livestock, organic cost share program, coronavirus food assistance program, and non-insured assistance program. To account for cases where an individual producer received multiple program payments, all duplicated addresses were removed.

All 6,238 program recipients of the identified programs were placed on our mailing list. Each of these producers received a letter with a URL and a QR code to access the survey on the QuestionPro platform and a unique six-digit code to open it. A reminder postcard was mailed two weeks later. The survey was open from September 26 to October 27, 2024. A total of 392 respondents entered the survey, yielding a response rate of 6.4%. To help ensure that only current cattle producers were included in the analysis, an additional screening question about whether respondents raised beef cattle was included in the survey. No compensation was provided for participating in the survey. The Institutional Review Board of South Dakota State University approved the study before data collection (IRB-2024–103). All participants provided implied consent by clicking a “Continue” button to enter the survey after reading a consent statement.

### Measures

The survey included questions that measured the use of established cattle vaccines (0–1 scale) and the type of vaccine (choose all that apply), concern about the impact of HPAI on their herd (1–4 scale), and the likelihood of using a cattle vaccine for HPAI (1–5 scale). Questions about herd size, primary source of income, HPAI outbreak awareness, and the frequency of veterinarian consultation were included, together with questions measuring age, gender, education, and personal COVID-19 vaccine uptake. The variables we measured in the survey were based on the existing literature [12,15]. An attention check question was also included. We report the full text of all questions and variable transformations in the appendix.

### Analysis

Participants who indicated not raising beef cattle for commercial purposes exited the survey. Participants who did not answer the attention check question correctly were not included in analysis. We described the data and then used binary logistic regression to determine the associations with regular vaccine use and ordered logistic regression on attitudes toward using a bird flu vaccine [16]. All variables described above were entered simultaneously as independent variables in both models, except for the concern about the bird flu variable that was included only in the second model. We reported adjusted odds ratios with 95% confidence intervals. Case-wise deletion was used to handle missing data. Goodness of fit was assessed with McFadden’s R-square, variance inflation factor tests were performed to test for multicollinearity, and Brant’s test of proportional odds was used to validate the parallel line assumption. We used STATA 18 for all data management and analysis [17].

## Results

We received 392 responses, with 339 current beef producers retained for analysis. The average age of participants was 53 years, 86% were men (n = 286), and 42% (n = 138) received at least a 4-year college degree. About 53% (n = 178) of participants indicated that raising beef cattle was the household’s primary source of income. Almost 45% (n = 148) of respondents indicated not receiving any COVID-19 vaccination, 21% (n = 71) completed the initial series, 17% received a booster dose (n = 57), 10% received multiple booster doses (n = 34), and 7% (n = 22) declined to answer. 292 participants answered the attention check question correctly.

Regarding herd size, 11% (n = 36) of participants reported having fewer than 50 head of cattle, 49% (n = 163) reported 51–200 head, 28% (n = 92) reported 201–500 head, 11% (n = 37) reported more than 500 head, and 2% (n = 6) of participants declined to answer. Over 20% (n = 68) of respondents consult with a veterinarian more than once a month, 51% (n = 169) consult with a veterinarian every couple of months, 19% (n = 64) consult twice a year, 9% (n = 29) yearly, and 1% (n = 4) never consult with a veterinarian.

More than 94% (n = 315) of participants indicated regularly vaccinating their cattle. Of those participants, 99% (n = 311) used reproductive vaccines for calves, 78% (n = 247) used reproductive vaccines for cows and heifers, 45% (n = 142) used scours vaccines, 37% (n = 117) used anthrax vaccines, and 13% (n = 41) reported using other vaccines. About 20% (n = 66) of all respondents were not at all concerned about the impact of bird flu on their cattle, 54% (n = 178) were not very concerned, 24% (n = 80) were somewhat concerned, and 2% (n = 8) were very concerned. About 23% (n = 77) of respondents were very unlikely to vaccinate their cattle for bird flu once a vaccine becomes available, 19% (n = 62) were somewhat unlikely, 34% (n = 113) were neither likely nor unlikely, 19% were somewhat likely (n = 64), and 5% (n = 16) were very likely. While not included in our modeling, but critical for the interpretation of the results given the potential threat HPAI poses, 88.2 percent of respondents were aware that bird flu had been found in the state’s dairy herd.

Regression results for both models are presented in Table 1. The results in the left column show the results for the use of established cattle vaccinations. The only statistically significant result was with the frequency of veterinarian consultation (aOR: 2.36, 95% CI: 1.36–4.12). The associations with other variables were not significant, including COVID-19 vaccination status (aOR: 1.45, 95% CI: 0.76–2.74). The McFadden’s R-squared value was 0.11. The results in the right column showed that the likelihood of using a hypothetical bird flu vaccine for cattle were associated with the producer COVID-19 vaccination status (aOR: 1.78, 95% CI: 1.41–2.26), frequency of veterinarian consultation (aOR: 1.40, 95% CI: 1.05–1.87), and concern about the impact of bird flu on their herd (aOR: 4.45, 95% CI: 3.07–6.45). The McFadden’s R-squared value was 0.13, suggesting good model specification. The R-squared values were consistent with benchmarks for social science research and compatible with similar studies [15]. In both models, variance inflation factor tests (VIF scores) showed very low levels of collinearity, and none of the independent variables were significant in Brant’s test of proportional odds.

**Table 1:** Predictors of vaccine use and vaccine attitudes among beef producers.

	Use of established cattle vaccines			Attitudes toward using bird flu vaccine for cattle		
	aOR	*p*	95% CI	aOR	*p*	95% CI
COVID-19 vaccination status	1.45	0.26	[0.76,2.74]	1.78	<0.01	[1.41,2.26]
Age	1	0.9	[0.96,1.04]	0.99	0.5	[0.98,1.01]
Male	1.66	0.5	[0.38,7.23]	1.05	0.89	[0.54,2.06]
Education	1.14	0.59	[0.72,1.80]	0.84	0.07	[0.70,1.02]
Primary income	1.25	0.76	[0.30,5.15]	0.98	0.94	[0.57,1.69]
Herd size	1.11	0.82	[0.46,2.65]	0.96	0.82	[0.68,1.35]
Veterinarian consultation	2.36	<0.01	[1.36,4.12]	1.4	0.02	[1.05,1.87]
Concern about bird flu				4.45	<0.01	[3.07,6.45]
Pseudo r-square	0.11			0.13		
Observations	260			259		

For ease of interpretation, we estimated predicted probabilities of respondents answering “somewhat likely” or “very likely” on the hypothetical use of an HPAI vaccine at varying levels of their personal COVID-19 uptake and frequency of consultation with a veterinarian (model 2). These are presented in Tables 2 and 3. Respondents who were unvaccinated for COVID-19 had a probability of answering “somewhat likely” of 0.097, whereas the probability for the same with a respondent who was fully vaccinated and had multiple boosters was 0.337. For respondents who were unvaccinated or fully vaccinated with multiple boosters, the probabilities of answering “very likely” were 0.016 and 0.084, respectively. A marked increase in both cases. The effects of frequency of consultation with a veterinarian had very similar positive associations. Respondents who never consult with veterinarians had a 0.070 probability of answering “somewhat likely” and 0.011 of answering “very likely” on the uptake of a hypothetical HPAI vaccine. For those who indicated they consult with a veterinarian monthly, the probabilities of the same were 0.212 and 0.041 respectively.

**Table 2:** Predicted probabilities of hypothetical HPAI vaccine use by COVID-19 vaccination status.

	Never	First Series	One Booster	Multiple Boosters
Somewhat Likely	0.097	0.158	0.240	0.337
Very Likely	0.016	0.028	0.049	0.084

**Table 3:** Predicted probabilities of hypothetical HPAI vaccine use by frequency of veterinarian consultation.

	Never	Yearly	Twice/Year	Every Few Months	Monthly
Somewhat Likely	0.070	0.095	0.126	0.166	0.212
Very Likely	0.011	0.015	0.021	0.030	0.041

## Discussion

We found very high use of established cattle vaccines, low concern about the impact of bird flu on cattle, and low likelihood of vaccinating cattle for bird flu once a vaccine becomes available.

First, the use of established vaccines was very high among our sample. The high level of vaccine deployment in the cow-calf operators surveyed could be affected by several different reasons. The cattle diseases outlined above are considered ubiquitous in cow-calf herds regardless of their geographic location, breed type, or use (e.g., producing seedstock vs. commercial calves). All of them represent conditions for which vaccines have been available for many years (in the case of blackleg vaccines, at least 100 years), therefore their safety and effectiveness have been observed over multiple generations of producers. Furthermore, the role of veterinarians in providing and recommending these products is important, as cattle producers have traditionally put a high degree of trust in those professionals.

These results are consistent with previous survey results in cow-calf producer vaccine use [18]. For example, a 2017–2018 survey of cow-calf operations in Canada found varied vaccination rates for different diseases, but at least 80% used at least one vaccination [19]. A 2008 Netherlands-based study found a mean 73% vaccination rate for cattle among commercial livestock farmers and hobby holders [20]. In the United States, 93% of cow-calf producers surveyed in 2016 had a vaccination plan [21] while the USDA Animal Plant Health Inspection Service reported that 75% of cow-calf producers used vaccines in 2017 [22]. In the latter survey, herd size significantly affected vaccination rates, where herds > 50 cows reported a vaccination rate greater than 86%. In our study, 94% of cow-calf producers reported vaccinating their cattle. Furthermore, herd sizes were greater than 50 cows for 89% of respondents to this survey.

Second, this survey revealed a low degree of concern from cattle producers about the effects that the HPAI virus may have on their animals. At the time of the survey, HPAI had not been identified in beef herds. It is possible that surveyed producers consider the virus a problem only in poultry and dairy farms due to their more intensive management and confinement, and lack of previous reports in beef cattle. Throughout the recent history of cow-calf production, there have been fewer emerging and novel infectious disease pathogens affecting those operations compared to swine and poultry operations. It is possible that these producers do not feel their operations are particularly at risk from new pathogens, having dealt with the same reproductive, respiratory, and environmental cattle diseases over multiple generations.

This lack of concern for the disease is likely an important factor affecting the low likelihood of these producers adopting a vaccine against HPAI in their own animals. However, the novel, possibly experimental, nature of an HPAI vaccine, having not been a time-tested disease intervention compared to the current beef cattle vaccine programs, may also be a factor.

With some animal conditions that are considered foreign animal diseases by the USDA, the use of certain vaccines against those diseases within a country confers that country with the same internationally recognized disease status as if the actual disease agent was present in the country’s animals. This fact has hampered the development and adoption of poultry vaccines against HPAI. It may be possible that some of these cattle producers considered that fact as a reason they would resist the use of an HPAI vaccine in their cattle.

Third, we also found that the frequency of veterinarian consultation was a strong predictor of cattle vaccine use and cattle vaccine attitudes. Scholars have shown the important role trust in veterinarians and veterinarian consultation plays in cattle vaccination decisions [15,23–25].

Fourth, the COVID-19 vaccination rate among our participants was low. It’s been documented that rural counties had lower COVID-19 vaccination rates than urban counties [7]. People employed in farming had lower COVID-19 vaccination rates than most other professions [8]. A study reported that veterinarians were reluctant to discuss COVID-19 vaccines with animal owners during routine visits, partly due to a concern of hostility toward COVID-19 vaccines among the population [26]. Many residents of rural areas viewed COVID-19 vaccination mandates as an infringement on their freedom [27]. Our results are in line with their findings.

### The relationship between human and cattle vaccination

The findings also pointed towards a relationship between human and animal vaccination. During the COVID-19 pandemic, some people developed negative attitudes toward COVID-19 vaccines and declined vaccination. Research indicates that these negative attitudes toward COVID-19 vaccines are affecting attitudes toward other vaccines. Evidence exists that people who did not get a COVID-19 vaccination or a booster are less likely to receive a flu vaccination [12] and other vaccines [11,28]. Following the start of the COVID-19 pandemic, the number of reports submitted to the Vaccine Adverse Event Reporting System about adverse events related to Measles, Mumps, and Rubella vaccination increased [29].

There is both direct and indirect evidence suggesting a negative impact of the COVID-19 pandemic on animal vaccination. According to one study, veterinarians in the United States and Canada reported an increased reluctance of pet owners to vaccinate their pets since the outbreak of COVID-19 [30]. Veterinarians reported noticing a relationship between the presence of movement against mandatory vaccination for children in their community and vaccine resistant or concerned clients [31]. A study in the United Kingdom reported that the number of pet vaccinations within one year of a veterinary consultation declined between 2016 and 2022 [32], suggesting a negative effect of the COVID-19 pandemic on animal vaccination.

Other studies surveyed pet owners following the outbreak of COVID-19. Preliminary evidence has linked owner COVID-19 vaccination status to pet vaccination uptake. Studies conducted in the United States showed that dog owners with negative attitudes toward human vaccines had negative attitudes toward canine vaccination, and that dog and cat owners with human vaccine hesitancy were less likely to vaccinate their pets [13,14]. A similar study conducted in Brazil reported that dog owners who were not fully vaccinated against COVID-19 were less likely to vaccinate their dogs [33]. These findings are consistent with a study conducted in Germany a year before the outbreak of COVID-19 that reported dog owners who received a tetanus vaccination within the past ten years were more likely to vaccinate their dogs [34].

Our paper added to this growing body of scholarship by looking at beef producers and cattle vaccination, which has been understudied. The findings from regression analyses were mixed. We did not find a relationship between personal COVID-19 vaccination status and the use of established cattle vaccines. Almost all the participants have been using established vaccines for a long time, and those decisions predate the COVID-19 pandemic. The data did not show a relationship between COVID-19 vaccine hesitancy among cattle owners and the use of established vaccine protocols for beef cattle. Nevertheless, we found that those beef producers who did not receive a COVID-19 vaccination were less likely to vaccinate their cattle for bird flu in the future. The outbreak of bird flu among dairy cattle in South Dakota began in 2024, well after the COVID-19 pandemic. The results showed an association between personal COVID-19 vaccination status and the likelihood of using a new cattle vaccine in the future. This finding highlights the importance of the One Health approach and studying the connection between human and animal health [35].

Unlike the time-tested use of established vaccines, exploring the likelihood of using a hypothetical HPAI vaccine is instructive. A negative effect on the use of traditional vaccines would be unlikely, given the generations of use and the economic consequences of not vaccinating. However, absent the years of practice and information about a potential HPAI vaccine, producers’ personal use of vaccines such as COVID-19, is more likely to predispose them towards or against new vaccines for their stock.

### Implications of our findings for veterinary health and veterinary vaccines

Our findings show that the COVID-19 pandemic might have long-term consequences on veterinary medicine, and beef producers choosing not to vaccinate cows with newly developed vaccines is just one example. It is plausible that beef producers will look at any other new cattle vaccine this way, which could have serious consequences for animal health and the beef industry. It is reasonable to consider that cattle producer likelihood of using an HPAI vaccination can serve as an indication of their attitudes toward the use of any new vaccine in their animals, regardless of the specific disease. Having already affected a population of cattle, albeit dairy cattle, HPAI represents a possible threat to their own animals. Therefore, their responses regarding their likelihood of vaccination for this virus are likely better informed than if they were asked about a hypothetical disease.

Regardless of the underlying reasons, the reluctance of food animal producers to adopt new preventive medicine practices, including vaccines, could hamper the larger response to nationally important emerging or novel animal diseases. The concept of herd immunity states that when a critical mass of animals within a population is resistant against a specific disease, animals comprising the entire population enjoy a decreased risk of disease regardless of their individual resistance status. On the other hand, should vaccine hesitancy result in a resistance rate below that critical number, the entire herd (whether that be the entire US cattle population or that of a specific state or region) is at increased risk for disease, even those that are vaccinated [36].

Governmental, professional, and industry entities looking to maximize the uptake of vaccines for newly-emerged or -introduced animal diseases must be aware of the behavioral aspects and viewpoints of animal producers around vaccination – particularly when it comes to new vaccines. Messaging to encourage the uptake of new vaccines in animal populations may need to be informed by approaches taken by public health messengers surrounding recent instances of vaccine hesitancy against human pathogens such as SARS-CoV2. In those cases, the use of trusted individuals within specific communities proved valuable. In the case of encouraging the use of cattle vaccines, entities might consider a similar approach using testimonials from trusted local professionals, such as veterinarians, or respected leaders within the cattle production industry.

### Limitations

Despite our efforts at constructing a representative sample, there are some issues that may affect our results. While the response rate of 6.4% is on par with other studies that utilize mailed invitations for online surveys [37], this is lower than other more costly methods that compensate respondents or send more frequent reminders [38]. The low response rate might raise questions about the potential for self-selection bias and generalizability of the findings. Producers with low trust in government or low trust in experts are less likely to vaccinate and participate in scientific studies like ours. Even though we were careful how we described the research in the invitation letter – not mentioning their own vaccination, it is possible that the beef producers who do not use vaccines on their livestock were underrepresented in our sample, either refusing to participate in the survey altogether upon reading the invitation or exiting the survey when the questions of their vaccination practices on their livestock or personal vaccination status appeared. The extent of adoption when the HPAI vaccine becomes available might be lower than reported here. Therefore, the results should be viewed as exploratory rather than confirmatory, and a starting point for future confirmatory research.

We are sensitive to these concerns, yet our findings about the established vaccines are in line with recently conducted surveys of cow-calf producers in the United States [39] and Canada [40]. Moreover, while our data collection approach led to a lower response, it also ensured high data quality. Studies based on data from surveys fielded on online crowdsourcing platforms often face questions about data quality due to the presence of inattentive participants. Impersonation is also a concern to data quality, particularly in studies targeting hard-to-recruit population groups, which provide higher compensation, and thus create greater incentive for fraud. In our study, we had full control of every step in the data collection process and are confident that our participants are beef producers from South Dakota. The use of physical mail to distribute study invitation letters and the use of unique access codes helped to recruit a representative sample and mitigate bias. Still, future work should consider compensation, more numerous reminders, partnering with professional organizations, and other measures to boost response rates.

We also acknowledge that our study explored the likelihood of using a hypothetical vaccine, which could lead to questions about the population-level implications of our findings. In the absence of an approved HPAI vaccine for cattle, all research about HPAI vaccine for cattle, both in veterinary and social sciences, is hypothetical [41]. Herd immunity to HPAI in beef cattle depends upon many factors, including the transmissibility of the virus, immunogenicity, and duration of the vaccine and natural infection, in addition to the level of vaccine use [42]. Our data captured the likelihood of using a hypothetical vaccine, which is a fundamental prerequisite for achieving herd immunity to this virus. This study provides foundation for future research, which will transition from investigating the likelihood of using a hypothetical vaccine to actual adoption behavior after the vaccine becomes available.

Finally, we encourage scholars to continue investigating the relationship between producer COVID-19 vaccination status and cattle vaccination practices. We only measured personal COVID-19 vaccination status and not attitudes toward COVID-19 vaccines. While these concepts are closely related, it is possible that some of our participants who received a COVID-19 vaccination do not have positive attitudes toward COVID-19 vaccines. We recommend that future studies contain both measures. Our data came from a single state, and while our participants were representative of beef cattle producers from states with cattle affected by HPAI, we encourage scholars to conduct multi-state studies. Our study was based on data from a cross-sectional survey, and we were able to identify an association between our variables of interest. Observational studies are limited in that they are not well suited for identifying causal relationships and to establish that negative attitudes toward COVID-19 vaccines precede the likelihood of using HPAI vaccines for cattle. We urge researchers to conduct longitudinal and experimental studies to further explore the relationship between producers’ personal vaccination decisions and cattle management practices. While these research designs require more resources, they are better suited to uncover inferences about COVID-19 spillover effects.

## Supporting information

S1 File Appendix. (DOCX)
