# Supplementary material for: The relationship between personal COVID-19 vaccination decisions and cattle vaccination practices: Evidence from a survey of South Dakota beef producers
Source: PLoS One. 2026 May 18;21(5):e0349234. doi: 10.1371/journal.pone.0349234 (PMC13183190; doi:10.1371/journal.pone.0349234)
Supplement: S1 File — (DOCX) [file pone.0349234.s001.docx]

**Supplementary Appendix**

**Survey Questions**

“Do you raise beef cattle for commercial purposes?”

- Yes, No

“Approximately how large is your cow herd?”

- Fewer than 50 head, 51-200 head, 201-500 head, More than 500 head, Prefer not to answer

“Is raising cattle your household's primary source of income?”

- Yes, No

“What was your age on your last birthday?”

“What is the highest level of education you have completed?”

- Some high school, High school diploma, Some college, Associate's degree (2-year), Bachelor's degree, Graduate degree

“What is your COVID-19 vaccination status?”

- Never vaccinated, Initial series completed , Initial series completed and boosted, Initial series completed and received multiple boosters, Prefer not to answer

“Researchers found that participants sometimes answer survey questions without reading them carefully, which can affect data quality. Please select Strongly agree to show that you are paying attention to this survey.”

- Strongly agree, Agree, Disagree, Strongly disagree

“How often do you consult with a veterinarian for care of your livestock?”

- Monthly or more often, Every couple months, Twice a year, Yearly, Never

“Do you regularly vaccinate your cattle?”

- Yes, No

[IF YES] “Which vaccines do your cattle regularly receive? (select all that apply)”

- Reproductive vaccines for cows and heifers (e.g. pre-breeding vaccines), Respiratory vaccines for calves (e.g. branding/turnout, pre-weaning, weaning vaccines), Scours vaccines for cows and heifers, Anthrax vaccines prior to grazing season

“How concerned are you that HPAI ("bird flu") will affect your own beef herd?”

- Very concerned, Somewhat concerned, Not very concerned, Not at all concerned

“If a bird flu vaccine for cattle becomes available, how likely are you to vaccinate your cattle?”

- Very likely, Somewhat likely, Neither likely nor unlikely, Somewhat unlikely, Very unlikely

**Variable Transformations**

Herd size question- ‘prefer not to answer’ answers were dropped

COVID-19 vaccination status question- ‘prefer not to answer’ answers were dropped
